# Rare Copy Number Variants Observed in Hereditary Breast Cancer Cases Disrupt Genes in Estrogen Signaling and *TP53* Tumor Suppression Network

**DOI:** 10.1371/journal.pgen.1002734

**Published:** 2012-06-21

**Authors:** Katri Pylkäs, Mikko Vuorela, Meeri Otsukka, Anne Kallioniemi, Arja Jukkola-Vuorinen, Robert Winqvist

**Affiliations:** 1 Laboratory of Cancer Genetics, Department of Clinical Genetics and Biocenter Oulu, Oulu University Hospital, University of Oulu, Oulu, Finland; 2 Laboratory of Cancer Genetics, Institute of Biomedical Technology/BioMediTech, University of Tampere and Fimlab Laboratories, Tampere, Finland; 3 Department of Oncology, Oulu University Hospital, University of Oulu, Oulu, Finland; University of Washington, United States of America

## Abstract

Breast cancer is the most common cancer in women in developed countries, and the contribution of genetic susceptibility to breast cancer development has been well-recognized. However, a great proportion of these hereditary predisposing factors still remain unidentified. To examine the contribution of rare copy number variants (CNVs) in breast cancer predisposition, high-resolution genome-wide scans were performed on genomic DNA of 103 *BRCA1*, *BRCA2*, and *PALB2* mutation negative familial breast cancer cases and 128 geographically matched healthy female controls; for replication an independent cohort of 75 similarly mutation negative young breast cancer patients was used. All observed rare variants were confirmed by independent methods. The studied breast cancer cases showed a consistent increase in the frequency of rare CNVs when compared to controls. Furthermore, the biological networks of the disrupted genes differed between the two groups. In familial cases the observed mutations disrupted genes, which were significantly overrepresented in cellular functions related to maintenance of genomic integrity, including DNA double-strand break repair (*P* = 0.0211). Biological network analysis in the two independent breast cancer cohorts showed that the disrupted genes were closely related to estrogen signaling and *TP53* centered tumor suppressor network. These results suggest that rare CNVs represent an alternative source of genetic variation influencing hereditary risk for breast cancer.

## Author Summary

Although genetic susceptibility to breast cancer has been well-established, the majority of the predisposing factors still remain unidentified. Here, we have taken advantage of recent technical and methodological advances to examine the role of a new class of genomic variation, rare copy number variants (CNVs), in hereditary predisposition to breast cancer. By examining 103 *BRCA1/2* and *PALB2* mutation negative familial and 75 young breast cancer cases, together with 128 geographically matched healthy female controls, we show that the frequency of rare CNVs is increased in cases when compared to controls and that the genes disrupted in individuals of specifically the two case groups are closely related to estrogen signaling and *TP53* centered tumor suppressor network. The variety of disrupted genes belonging to these networks underscores that diverse mechanisms are likely to be relevant to breast cancer pathogenesis. The current results warrant the investigation of rare CNVs as new susceptibility factors in other cancer types as well.

## Introduction

Breast cancer is the most common malignancy affecting women. It is a complex disease with a well-established genetic component [1]; however, most of the familial and young breast cancer cases still remain unexplained by inherited mutations in the known susceptibility genes [2]. Multiple genome-wide association studies (GWAS) have identified several breast cancer associated single nucleotide polymorphisms (SNPs), but these have only modest effect sizes and explain much less of the heritability than originally anticipated [3]. Consequently, the contribution of rare variants with moderate to even high disease penetrance is now beginning to be more widely accepted. With the exception of some specific founder mutations, these rare variants are individually infrequent, and even specific to single cases or families. Much of the work with rare genomic variants has been conducted through candidate gene re-sequencing studies mainly concentrating on DNA damage response genes, Fanconi anemia/BRCA pathway genes in particular, and their coding region variations [2]. However, rare genomic microduplications and microdeletions, also known as structural variants or copy number variants (CNVs), could represent an alternative class of genetic variation responsible for increased cancer risk.

Recent reports have suggested a role for genomic structural variants in susceptibility to various diseases, particularly neurodevelopmental disorders [4], [5]. Association of common CNVs with breast cancer susceptibility has been ruled out by a recently performed large case-control study [6], but the contribution of rare CNVs still remains poorly explored. As alleles in this variation class will be individually rare, the studies remain statistically underpowered to identify any specific loci involved, but the overall involvement can be tested by comparing the collective frequency of rare variants in cases with that in controls [5]. Moreover, the functional profiling of the disrupted genes will have a potential to reveal biological processes, which when defective could predispose to breast cancer. The known susceptibility genes are already considered to cause cancer predisposition through different mechanisms. Whereas *BRCA1* and *BRCA2* function in DNA repair [7], other high-risk susceptibility genes, *TP53* and *PTEN*, participate in cell cycle control and regulation of cell proliferation [8], [9].

Here we have examined whether rare CNVs throughout the genome display an increased frequency in familial and young breast cancer cases when compared to healthy controls, and whether the biological pathways or processes, to which the disrupted genes relate to differ between the groups. Our results provide evidence that rare CNVs contribute to breast cancer susceptibility and that the disrupted genes are closely related to the *TP53* tumor suppression network and to estrogen signaling.

## Results

### Rare CNV discovery in breast cancer cases and controls

Genome-wide scans for structural variants were performed on 103 familial breast cancer cases and 128 controls, using high-resolution Illumina HumanOmni1-Quad BeadChips. Stringent quality control criteria were applied to ensure that ascertainment of CNVs was consistent between cases and controls. The frequencies of common CNVs were monitored in both groups, and their frequency did not significantly differ (mean 9.7 CNVs for cases and 9.13 CNVs for controls). Rare variants were defined as those that did not overlap over 60% with the common CNVs in Toronto Database of Genomic Variants, and all CNVs fulfilling the rare variant criteria were confirmed by independent method. In the studied 231 subjects we observed 65 microdeletions and microduplications, ranging in size from 25 kb to 612 kb. In cases, there were 15 deletions (mean length 123 kb, median 61 kb) and 20 duplications (mean 216 kb, median 173 kb), whereas in controls 14 deletions (mean 146 kb, median 133 kb) and 16 duplications (mean 242 kb, median 186 kb) were observed.

Among familial breast cancer cases the total number of rare CNVs was slightly higher than in controls: their proportion was also higher when only considering those rare CNVs involving genes, and those directly disrupting genes. This trend stayed the same when analyzing the independent young breast cancer cohort of 75 patients (Table 1). The difference was most profound when considering CNVs disrupting genes and restricting the analysis to variants not shared between cases and controls. Familial cases showed almost twice, and young breast cancer cases 1.5 times the number of rare CNVs compared to controls, but none of the differences were statistically significant. The genes within each rare CNV locus were identified (Tables S1, S2 and S3), and functions and pathways of the involved genes (Table S1) were assessed by using the Ingenuity Pathway Analysis (IPA) classification system.

**Table 1:** Proportion of rare CNVs in breast cancer cases and controls.

		All observed rare CNVs			Observed rare CNVs, not shared^a^		
Subjects	*n*	All	Involving genes^b^	Disrupting genes^c^	All	Involving genes^b^	Disrupting genes^c^
Familial BC cases	103	0.34 (35/103)	0.29 (30/103)	0.24 (25/103)	0.25 (26/103)	0.20 (21/103)	0.17 (17/103)
Young BC cases	75	0.32 (24/75)	0.24 (18/75)	0.23 (17/75)	0.23 (17/75)	0.15 (11/75)	0.13 (10/75)
Controls	128	0.23 (30/128)	0.21 (27/128)	0.16 (20/128)	0.16 (21/128)	0.14 (18/128)	0.09 (12/128)

BC = breast cancer.

a Observed only in cancer cases, or only in controls.

b The genomic loci has annotated genes.

c Gene disruptions include rare CNVs having breakpoints within the genes or promoter regions, and rare CNVs which delete the involved genes entirely.

### Genes disrupted in familial cases show enrichment in genomic integrity maintenance functions and diabetes

Analyses were restricted to genes, which were either disrupted by the breakpoints or deleted entirely, as mutations disrupting only part of the gene are likely to have biological consequences, and entirely deleted genes in the case of tumor suppressors follow the rationale of Knudson's two hit model or haploinsufficiency [10]. Only a few of the disrupted genes were part of known canonical pathways, and neither cases nor controls showed significant increase in any of them. The genes disrupted in familial cases showed, however, a significant overrepresentation in functions involving the maintenance of genomic integrity (Table 2), whereas no particular functions were overrepresented among controls. Three of the genes disrupted in cases were directly involved in double-strand break (DSB) repair signaling: BLM participates in BRCA1-mediated DNA damage response [11], RECQL4 is involved in DNA replication and DSB repair [12], and DCLRE1C operates in DSB repair by non-homologous end joining [13]. Both BLM and RECQL4 are RecQ family DNA helicases with an integral role in the maintenance of genomic stability. Their defects result in recessive cancer predisposition syndromes, Bloom and Rothmund-Thompson syndrome, respectively [14], [15]. *DCLRE1C* encodes ARTEMIS, which is essential for V(D)J recombination. Biallelic mutations result in severe combined immunodeficiency (SCID), in which lymphoma has been described [16]. Curiously, the currently observed *DCLRE1C* allele is one of the most frequent mutations reported among SCID patients. This null allele comprises a gross deletion of exons 1–4 and the adjacent *MEIG1* gene and results from homologous recombination of *DCLRE1C* with the pseudo-*DCLRE1C* gene, located 61.2 kb upstream [17]. Based on their biological functions *BLM*, *RECQL4* and *DCLRE1C* all represent attractive susceptibility genes, although to date clearly deleterious, breast cancer related mutations have not been reported in any of them. Although not significantly overrepresented, it should be noted, however, that another DNA repair gene, *MCPH1*, was found to be disrupted in one of the studied controls. MCPH1 is an early DNA damage responsive protein, the dysfunction of which leads to recessive primary microcephaly without any reported malignancies [18]. The observed CNV deletes exon 13 and is predicted to lead to out of frame translation of the last exon, number 14, thereby disrupting one of the three BRCT domains of MCPH1. The carrier was still healthy at the age of 59 years, supporting the previous notion that all DNA damage response gene deficiencies do not necessarily predispose to malignancy.

**Table 2:** Molecular and cellular functions, and diseases and disorders overrepresented among the genes disrupted in familial breast cancer cases.

Molecular and cellular functions	*P*-values^a^	Genes involved
Organization of chromosomes	0.0133	*BLM*, *DCLRE1C*
Maintenance of telomeres	0.0133	*BLM*, *DCLRE1C*
Repair of DNA	0.0178	*RECQL4*, *BLM*, *DCLRE1C*
Double-stranded DNA break repair	0.0211	*BLM*, *DCLRE1C*
Quantity of *corpus luteum*	0.00367	*CASP3*, *ESR2*
**Diseases and disorders**		
Diabetes mellitus	0.000268	*ACSL1,ANKS1B,ARHGAP39,BLM,CASP3,ESR2,KCNIP4,KLHL1,MARCH6,MLF1IP,RBFOX1,STRN,SYNE2*

No particular functions were overrepresented among controls.

a Statistically significant false discovery rate (FDR) adjusted *P*-values; correction for multiple testing was done using the Benjamini-Hochberg method.

The genes disrupted in familial cases were also highly overrepresented among genes connected to diabetes mellitus (*P* = 0.000268); this connection was mediated mainly through SNP associations observed in GWAS [19]. This overrepresentation was also seen in the young breast cancer cohort (*P* = 0.0246), but not in controls. Of the 16 diabetes associated genes 6 were under β-estradiol regulation.

### Network analysis reveals TP53 and β-estradiol centered networks in breast cancer cases

**Figure 1:**
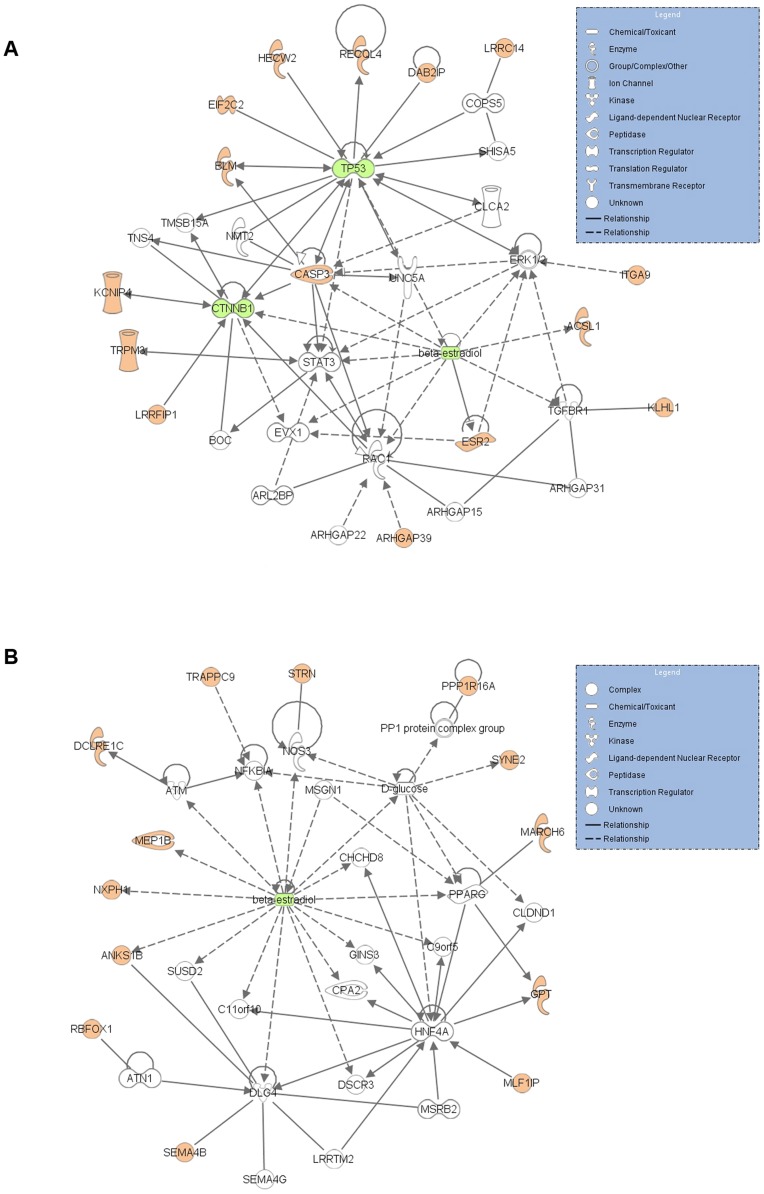
Indication of dysfunction of *TP53* and β-estradiol centered network in the studied breast cancer cases. IPA was used to identify the connection between the genes disrupted in all cases (both familial and the cohort consisting of young breast cancer patients). The analysis identified two networks with (A) *TP53*, β-estradiol and *CTNNB1* (in green) occupying the central positions, and (B) β-estradiol (in green) occupying the central position. Genes disrupted in breast cancer cases are coloured with red. Solid lines indicate direct molecular interaction and dashed lines indicate indirect molecular interaction.

The strict pathway-based approach has several limitations as the function of many genes is currently unknown and cannot be assigned to any predetermined pathways [20]. Consequently, we next analyzed IPA networks, which map the biological relationships of the uploaded genes. Curiously, analysis with familial cases revealed a network centered on *TP53* and β-estradiol (score 29). The same *TP53* and β-estradiol centered network was observed when analyzing genes disrupted in the young breast cancer cohort (score 28) (Figures S1 and S2). When analyzing both case cohorts together the network with the highest scores (35, 31) centered on *TP53*, β-estradiol and *CTNNB1* (encoding β–catenin, the oncogenic nuclear accumulation of which occurs in several malignancies, including breast cancer [21]) and the other around β-estradiol (Figure 1, Table 3). Neither the *TP53* nor β-estradiol centered network was observed in controls, strongly arguing in favour of the possibility that dysregulation of these networks is disease related.

**Table 3:** Genes disrupted or deleted entirely in breast cancer cases and involved in *TP53* and β-estradiol centered network.

Gene	Aberration type	Involved exons^a^	Predicted consequence to transcript^b^
*BLM*	disruption	promoter dup	unknown
*EIF2C2*	disruption	ex2-ex18,3′UTR dup	unknown
*HECW2*	disruption	promoter, ex1 dup	unknown
*RECQL4*	deletion	entire gene	null allele
*DAB2IP*	disruption	promoter, ex1 del	null allele
*LRRC14*	deletion	entire gene	null allele
*ITGA9*	disruption	ex19-ex23 del	in frame deletion
*ACSL1*	deletion	entire gene	null allele
*KLHL1*	disruption	promoter, ex1 del	null allele
*ESR2*	disruption	ex2-ex9, 3′UTR dup	unknown
*ARHGAP39*	disruption	ex12-ex13, 3′UTR del	premature termination
*LRRFIP1*	disruption	promoter, ex1 dup	unknown
*CASP3*	deletion	entire gene	null allele
*TRPM3*	disruption	promoter, ex1 dup	unknown
*KCNIP4*	disruption	ex6-ex9, 3′UTR dup	unknown
*DCLRE1C*	disruption	promoter, ex1-ex4 del	null allele
*TRAPPC9*	disruption	promoter, ex1-ex3 dup	unknown
*STRN*	disruption	ex14-ex18, 3′ UTR dup	unknown
*PPP1R16A*	disruption	ex6-ex10, 3′UTR del	premature termination
*SYNE2*	disruption	ex50-ex114, 3′UTR dup	unknown
*MARCH6*	disruption	ex4-ex26, 3′UTR dup	unknown
*GPT*	deletion	entire gene	null allele
*MLF1IP*	deletion	entire gene	null allele
*SEMA4B*	disruption	ex3-ex15, 3′UTR dup	unknown
*RBFOX1*	disruption	ex11-ex13, 3′UTR dup	unknown
*ANKS1B*	disruption	promoter, ex1 dup	unknown
*NXPH1*	disruption	promoter, ex1-ex2 del	null allele
*MEP1B*	deletion	entire gene	null allele

Disruption = the gene is disrupted by the CNV breakpoints; deletion = the entire gene is deleted. del = partial gene deletion; dup = partial gene duplication.

a Based on human genome assembly 19 (February 2009).

b Although detailed effects of partial gene duplication to gene transcription are not clear, duplication have potential to disrupt transcription by several mechanisms, such as transcriptional read-through. This can occur by tandem duplication, where gene silencing can be induced by a partially duplicated (3′ deleted) version of the gene itself [54].

The *TP53* centered network appears to have obvious tumor suppressive function, as p53 itself is a key regulator in preventing cells from malignancy. Somatic *TP53* mutations occur frequently in human malignancies, and germline lesions associate with the cancer prone Li-Fraumeni syndrome [22]. In the studied breast cancer cases, six genes disrupted by the observed rare CNVs were directly linked to *TP53* (Figure 1A), and all encode proteins functioning in pathways with a potential role in malignancy prevention. Two of these, RECQL4 and BLM, were DNA damage response proteins. Network interactions were based on the repression of *RECQL4* transcription by p53 [23], and the requirement of BLM for p53 localization to stalled replication forks [24]. The other four interactions were based on direct binding of p53 with HECW2 [25], DAB2IP and EIF2C2 [26]; for *CASP3* p53 has been shown to increase its activation [27]. The *HECW2* disrupting allele was observed in two familial cases, whereas the others were all singletons (Table S1).

The other network indicated in both of the studied breast cancer case cohorts centered on β-estradiol (Figure 1A and 1B), which is the primary biologically active form of estrogen. Exposure to both exogenous and endogenous estrogens is a well-established risk factor for breast cancer, and disruptions in estrogen signaling and metabolism have a potential to affect this risk. The physiological effects of estrogens are mediated by their ability to alter the expression of their target genes. Estrogens play a key role in proliferation and differentiation of healthy breast epithelium, but also contribute to the progression of breast cancer by promoting the growth of transformed cells [28]. Many of the estrogen actions are mediated by intracellular estrogen receptors ESR1 and ESR2 [29]. The β-estradiol centered network consisted of several β-estradiol responsive genes, *ANKS1B* [30], *NXPH1*, *MEP1B* [31], *CASP3* [32] and *ACSL1* [31], whereas when separately tested in IPA none of the genes disrupted in controls were found to be under β-estradiol regulation. Of the network genes *ESR2*, *STRN* and *ANKS1B* exhibited recurrent disrupting alleles among cancer cases (Table S1), emphasizing their potential role in breast cancer predisposition.

## Discussion

The results from our high-resolution genome-wide scans for structural variants provide evidence that rare CNVs contribute to breast cancer susceptibility. When compared to controls, the studied breast cancer cases showed a slight but consistent increase in the frequency of rare CNVs. The difference was not as profound as seen in psychiatric disorder studies where the observed changes, typically involving large genomic regions and numerous genes, can have very severe effects on patients' phenotype and many of which are *de novo* mutations [4], [5]. However, in our study the biological networks affected by the disrupted genes differed between breast cancer cases and controls, supporting their role in cancer predisposition.

The genes disrupted in familial cases showed a significant overrepresentation in functions involving the maintenance of genomic integrity. This included DSB repair, which is consistent with the prevailing paradigm that defects in this pathway contribute to breast cancer predisposition [2]. The three DSB repair genes, *BLM*, *RECQL4* and *DCLRE1C*, disrupted in the case group all represent attractive breast cancer susceptibility genes. Moreover, IPA analysis demonstrated that the genes disrupted by rare CNVs in the studied breast cancer cases formed a network centered on *TP53* and β-estradiol, a notion confirmed in two independent cohorts. Both networks are coherent and biologically meaningful, and their identification through the used genome-wide approach provides strong evidence for a role in breast cancer predisposition.

*TP53* network genes encode proteins functioning in pathways with potential role in malignancy prevention, including DNA damage response and apoptosis [25], but also RNA interference [33]. They all represent attractive susceptibility genes, which could harbor also other cancer predisposing mutations; thus being excellent candidates for re-sequencing studies. Of the disrupted *TP53* network genes *DAB2IP* and *CASP3* were particularly interesting. *DAB2IP* is a member of the Ras GTPase-activating gene family and has been reported to act as a tumor suppressor. Inactivation of DAB2IP by promoter methylation occurs in several malignancies, including prostate and breast cancer [34], and it has been shown to modulate epithelial-to-mesenchymal transition and prostate-cancer metastasis [35]. *CASP3* is an apoptosis related gene, which encodes a member of a highly conserved caspase protease family, caspase 3. Caspases are key intermediaries of the apoptotic process, failure of which can lead to cancer [36]. Various molecular epidemiological studies have suggested that SNPs in caspases may contribute to cancer risk, and a common coding variant in caspase 8 has been associated with breast cancer susceptibility [36], [37]. Curiously, apoptosis is also one of the numerous genomic integrity maintenance functions of BRCA1. Caspase 3 has been reported to mediate the cleavage of BRCA1 during UV-induced apoptosis, and the cleaved C-terminal fragment triggers the apoptotic response through activation of BRCA1 downstream effectors [38]. The rare CNVs disrupting the *DAB2IP* and *CASP3* genes were both predicted to result in null alleles (Table 3).

For estrogen, there are multiple lines of evidence for its profound role in breast cancer development, and disruptions in estrogen signaling and metabolism have long been considered to affect breast cancer risk. The estrogen network was largely explained by the genes under β-estradiol regulation, but two of the disrupted genes, *ESR2* and *STRN*, had a more straightforward role in estrogen signalling. *ESR2* encodes the estrogen receptor β, which is one of the main mediators of estrogen actions within the cell [29]. It binds estrogens with a similar affinity as estrogen receptor α, and activates expression of estrogen response element containing genes [39]. *ESR2* has previously been suggested to harbor common breast cancer predisposing variants [40], [41], and ESR2 variation has been suggested to influence the development of breast cancer also by *in vitro* studies [42]. In contrast, striatin acts as molecular scaffold in non-genomic estrogen-mediated signaling [43]. It physically interacts with calmodulin 1 [44] and estrogen receptor α, and also forms a complex with protein phosphatase 2A, which also regulates the function of estrogen receptor α [45]. The identification of a recurrent deletion allele in *CYP2C19*, encoding an enzyme involved in estrogen metabolism [46] and with an increased frequency in familial cases (Table S2), further emphasizes the role of estrogen in breast cancer predisposition. One *CYP2C19* allele, CYP2C19*17, defining an ultra-rapid metabolizer phenotype, has previously been associated with a decreased risk for breast cancer. This suggests that increased catabolism of estrogens by CYP2C19 may lead to decreased estrogen levels and therefore reduced breast cancer risk [47]. Correspondingly, decreased activity of CYP2C19 through haploinsufficiency might potentially increase the risk of breast cancer. Curiously, based on their function both *ESR2* [40], [41] and *CYP2C19* [47] have long been considered strong candidate genes for breast cancer susceptibility. However, no structural variants have previously been reported in either of them, and it is possible that CNVs might represent a new class of cancer predisposing variation in both genes. Functionally relevant structural variants might be present also in other *CYP* genes that locate in gene clusters, like *CYP2C19* [48]. The clustering of similar genes increases the potential for unequal crossing-over between sister chromatids and thus for creation of CNV alleles.

The genes disrupted in both studied breast cancer cohorts were also significantly overrepresented among genes connected to diabetes mellitus. This unexpected result likely represents shared risk factors predisposing to both breast cancer and diabetes. Indeed, these two diseases have already been reported to share several non-genetic risk factors, including obesity and a sedentary lifestyle. The hormonal factors altered in diabetes include several hormonal systems that may also affect the development of breast cancer, including insulin, insulin-like growth factors, and other growth factors as well as estrogen [49], [50]. Our results support estrogen being the key link in the association between diabetes and breast cancer, as over one third of the diabetes associated genes in the two studied breast cancer cohorts were part of the β-estradiol network.

In conclusion, rare CNVs should be recognized as an alternative source of genetic variation influencing breast cancer risk. This notion is further supported by a recent study which also provided evidence for rare CNVs' contribution to familial and early-onset breast cancer [51]. The results from the current network analysis with two independent breast cancer cohorts provide strong evidence for the role of estrogen mediated signaling in breast cancer predisposition and reinforce the concept of *TP53* centered tumor suppression in the prevention of malignancy. The variety of disrupted genes belonging to these networks underscores that diverse mechanisms are likely to be relevant to breast cancer pathogenesis.

## Materials and Methods

### Subjects

The studied familial breast cancer cohort consisted of affected index cases of 103 Northern Finnish breast, or breast-and ovarian cancer families. 73 of the families were considered as high risk ones: 67 had three or more cases of breast cancer, potentially in combination with single ovarian cancer in first- or second-degree relatives, and 6 had two cases of breast, or breast and ovarian cancer in first- or second-degree relatives, of which at least one with early disease onset (<35 years), bilateral breast cancer, or multiple primary tumors including breast or ovarian cancer in the same individual. The remaining 30 families were indicative of moderate disease susceptibility, and had two cases of breast cancer in first- or second-degree relatives, of which at least the other breast cancer was diagnosed under the age of 50. The median at the age of diagnosis for the familial cases was 49 years (variation 26–89 years), and all families were negative for Finnish *BRCA1*, *BRCA2*, *TP53* and *PALB2* founder mutations [52].

The studied young breast cancer cohort consisted of 75 Northern Finnish patients that were diagnosed with breast cancer at or under the age of 40 (median 38, variation 25–40 years). These patients were unselected for a family history of the disease, and tested negative for Finnish *BRCA1*, *BRCA2* and *PALB2* founder mutations. This independent breast cancer cohort was collected as a validation group for the studied familial cases, based on the assumption that when a woman under the age of 40 years develops breast cancer, a hereditary predisposition may be suspected regardless whether there is a family history or not [53]. All biological specimens and clinical information of the familial and young breast cancer cases investigated were collected at the Oulu University Hospital, with the written informed consent of the patients. The geographically and ancestrally matched control group consisted of 128 anonymous cancer-free female Northern Finnish Red-Cross blood donors (median age at monitoring was 56, variation 50–66 years). Permission to use the above mentioned patient and control materials for studies on hereditary predisposition to cancer has been obtained from the Finnish Ministry of Social Affairs and Health (Dnr 46/07/98), and the Ethical Committee of the Northern-Ostrobothnia Health Care District (Dnr 88/2000+amendment). All genomic DNA samples analyzed derived from blood samples extracted using either the standard phenol-chloroform method, Puregene D-50K purification kit (Gentra, Minneapolis, MN, USA), or UltraClean Blood DNA Isolation Kit (MoBio, Carlsbad, CA, USA ) and no DNA samples from immortalized lymphoblastoid cell lines were used.

### CNV discovery with Illumina platform

CNV discovery for both the familial and young breast cancer cohort as well as for the healthy controls was performed by using Illumina HumanOmni1-Quad BeadChips (Illumina Inc., San Diego, CA, USA). This provides high-resolution coverage of the genome with over one million genetic markers, including those derived from the 1,000 Genomes Project and all three HapMap phases, and enables precise definition of the breakpoints. All samples included in the array had to pass the standard quality control (QC) measures, which included agarose gel runs to confirm the integrity of the DNA sample, and accurate concentration determination with three-step dilution measurements. To control the confounding effects resulting from the handling of the samples and subsequent CNV analysis, all cases and controls were given new IDs and were blindly analyzed without knowing their disease status. All samples were analyzed following the Illumina provided protocol in the same laboratory (Laboratory of Cancer Genetics, University of Oulu) with same arrays at the same period of time, with random places on the chip.

Samples were analyzed with GenomeStudio Genotyping module (Illumina) and Nexus Copy Number Discovery Edition 5.1 software (BioDiscovery Inc., El Segundo, CA, USA). Projects were created in GenomeStudio, and samples having Call Rates over 98% were transported to Nexus where samples with quality score <0.15 were passed on for further analysis. In order to obtain a high-quality CNV dataset, we restricted the analysis to CNVs called by two independent algorithms. In Nexus the SNP-FASST2 segmentation algorithm was used. The significance threshold was set to 1.0E-06, and +0.25 for gains and −0.25 for losses. The minimum number of probes needed for segment calling was set to 25, and minimum loss of heterozygosity length to 10 000 kb. Quadratic correction was used as a systematic correction of artifacts caused by GC content and fragment length. Samples passing all the QCs but showing over 50 copy number changes in Nexus were excluded. The sensitivity of detection in Nexus was evaluated by analyzing 11 samples containing known deletions/amplifications confirmed by independent methods, and all changes were detected under the parameters used. All observed CNVs had to be confirmed by Illumina cnvPartition 2.4.4 software, using a confidence level of over 50 in order to be included in the analysis: values of 50 or higher tend to reflect a region with high confidence. The breakpoints of the observed aberrations were defined using the information obtained from both Nexus and GenomeStudio, and CNVs that appeared to be artificially split by the algorithm were joined.

The focus of our interest was on rare duplications and deletions. Rare events were defined as those which were called by two independent algorithms and did not overlap over 60% with the common CNVs in the CNV track defined in Nexus, based on the Toronto Database of Genomic Variants (DGV). However, as the DGV database presents several known cancer susceptibility genes as containing polymorphic CNVs, each CNV not fulfilling the rare variant criteria were individually inspected before exclusion. As a result, we decided to include “common” CNV in the rare variant analysis if fulfilling all three of the following criteria: 1) the CNV disrupts the involved gene partially, or deletes it entirely, 2) affected gene is a known breast cancer susceptibility gene, or based on it biological function it is a highly likely breast cancer susceptibility gene, and 3) biallelic defects in the involved gene lead to a rare genomic disorder, indicating that the defective allele is highly unlikely to be polymorphism. This led to inclusion of three alleles disrupting the following genes: *RECQL4*, *MCPH1* and *DCLRE1C*. All “rare” events which were present at polymorphic frequencies in the pooled population of 250 cases and controls, except those that were specific or showed a clear enrichment in cancer cases, were excluded from further analyses.

All potential events of interest, rare CNV variants, were validated by another independent method, either by Affymetrix Genome-Wide Human SNP Array 6.0 platform (Affymetrix, Santa Clara, CA, USA) or quantitative real time PCR (qPCR). Affymetrix chip analysis was performed following all the QC measures recommended by the protocol, and Affymetrix CEL files were transported to Nexus for analysis with the SNP-FASST2 segmentation algorithm. Confirmation with qPCR was done with BioRad CFX96 using SsoFast EvaGreen Supermix (BioRad, Hercules, CA, USA). Samples with rare CNVs and at least 3 wildtype controls were analyzed in triplicate, and quantitation was done with CFX manager software (version 1.5) under gene expression analysis. *RAD50* and *CtIP* were used as reference genes.

### Statistical analyses

Rare variant carrier frequencies between cancer cases and controls were compared using Fisher's exact test. The frequency of common CNVs and the size of duplications and deletions was monitored both in cases and controls and tested for differences with Mann-Whitney U-test (PASW Statistics 18.0 for Windows, SPSS Inc., Chicago, IL, USA). All tests were two-sided and considered to be statistically significant with a *P*-value≤0.05.

### Network analysis and functional profiling

For pathway and biological function analysis, Ingenuity Pathway Analysis (IPA, http://www.ingenuity.com/) was used. The list of disrupted genes [defined as genes (including also their promoter region) disrupted by the breakpoints or deleted entirely, and not shared between cases and controls] were uploaded to IPA, which is an online exploratory tool with a curated database for over 20,000 mammalian genes and 1.9 million published literature references. Together with several databases, including Entrez Gene, Gene Ontology and GWAS database, IPA integrates transcriptomics data with mining techniques to predict and build up networks, pathways and biological function clusters. The software maps the biological relationships of the uploaded genes according to published literature included in the Ingenuity database. The output results are given as scores and *P*-values computed based on the numbers of uploaded genes in the cluster or network and the size of network or cluster in the Ingenuity knowledge database. Benjamini-Hochberg multiple testing correction *P*-values (to monitor the false discovery rate) were used to determine the probability that each biological function or overrepresentation in diseases is due to change alone. Scores for IPA networks are the negative logarithm of the *P*-value, and they indicate the likelihood of the genes analyzed in a network for being found together due to random chance. Scores 2 or higher have at least a 99% likelihood of not being generated by chance alone.

## Supporting Information

Figure S1 *TP53* and β-estradiol centered network in familial breast cancer cases. IPA was used to identify the connection between the genes disrupted in familial breast cancer cases. The analysis identified a network with *TP53* and beta-estradiol (in green) occupying the central positions. Genes disrupted in breast cancer cases are coloured with red. Solid lines indicate direct molecular interaction and dashed lines indicate indirect molecular interaction.
(JPG)

Figure S2 *TP53* and β-estradiol centered network in young breast cancer cases. IPA was used to identify the connection between the genes disrupted in young breast cancer cases. The analysis identified a network with *TP53* and β-estradiol (in green) occupying the central positions. Genes disrupted in breast cancer cases are coloured with red. Solid lines indicate direct molecular interaction and dashed lines indicate indirect molecular interaction.
(JPG)

Table S1 Novel rare CNVs in genomic DNA that delete or duplicate genes in breast cancer cases and controls.
(DOC)

Table S2 Novel rare CNVs in genomic DNA that delete or duplicate genes observed in both breast cancer cases and controls.
(DOC)

Table S3 Novel rare CNVs in genomic DNA that delete or duplicate genomic regions without annotated genes in breast cancer cases and controls.
(DOC)
