# Supplementary figures and images for: Rare Copy Number Variants Observed in Hereditary Breast Cancer Cases Disrupt Genes in Estrogen Signaling and TP53 Tumor Suppression Network
Source: PLoS Genet. 2012 Jun 21;8(6):e1002734. doi: 10.1371/journal.pgen.1002734 (PMC3380845; doi:10.1371/journal.pgen.1002734)

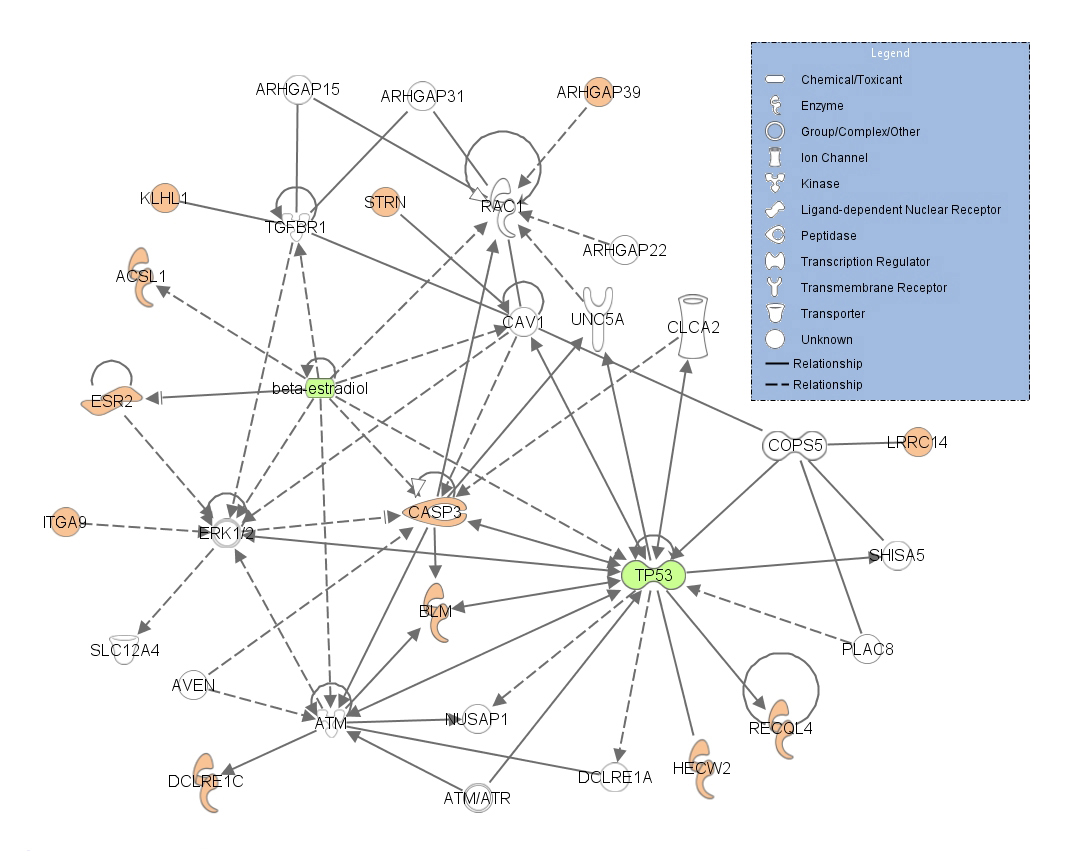

Supplement: Figure S1 — TP53 and β-estradiol centered network in familial breast cancer cases. IPA was used to identify the connection between the genes disrupted in familial breast cancer cases. The analysis identified a network with TP53 and beta-estradiol (in green) occupying the central positions. Genes disrupted in breast cancer cases are coloured with red. Solid lines indicate direct molecular interaction and dashed lines indicate indirect molecular interaction. (JPG) [file pgen.1002734.s001.jpg]

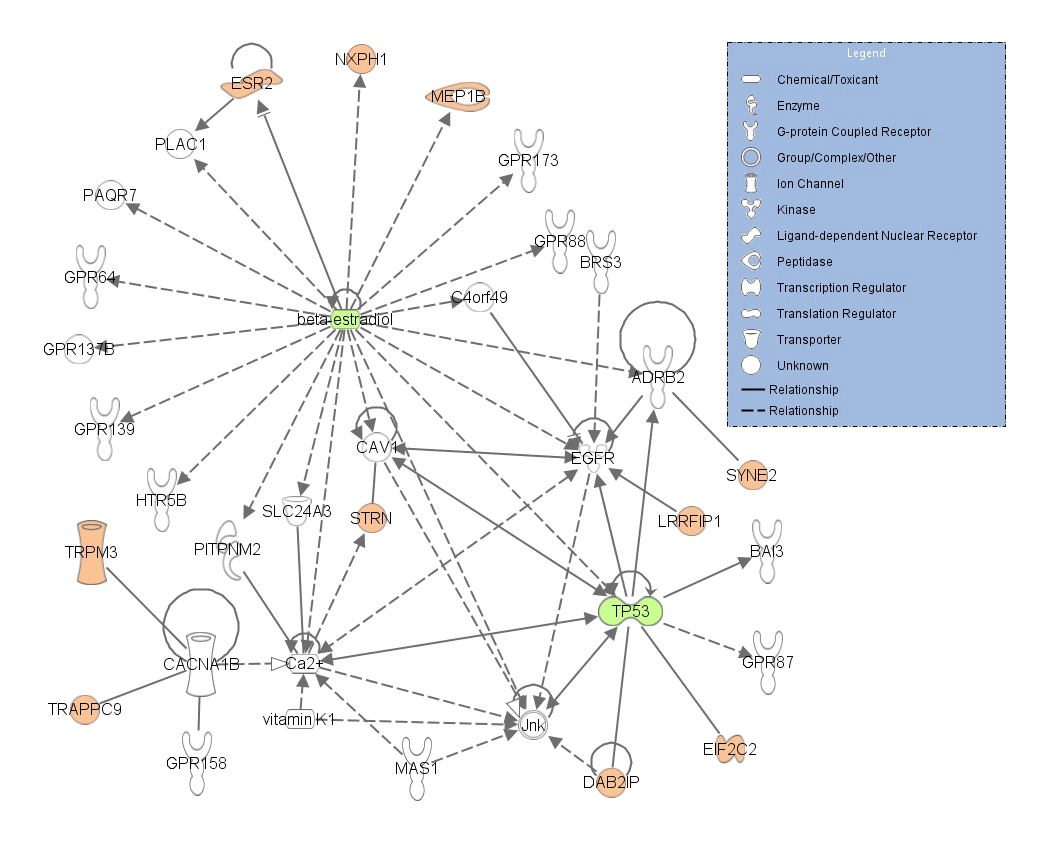

Supplement: Figure S2 — TP53 and β-estradiol centered network in young breast cancer cases. IPA was used to identify the connection between the genes disrupted in young breast cancer cases. The analysis identified a network with TP53 and β-estradiol (in green) occupying the central positions. Genes disrupted in breast cancer cases are coloured with red. Solid lines indicate direct molecular interaction and dashed lines indicate indirect molecular interaction. (JPG) [file pgen.1002734.s002.jpg]
