# Supplementary material for: Rare Copy Number Variants Observed in Hereditary Breast Cancer Cases Disrupt Genes in Estrogen Signaling and TP53 Tumor Suppression Network
Source: PLoS Genet. 2012 Jun 21;8(6):e1002734. doi: 10.1371/journal.pgen.1002734 (PMC3380845; doi:10.1371/journal.pgen.1002734)
Supplement: Table S1 — Novel rare CNVs in genomic DNA that delete or duplicate genes in breast cancer cases and controls. (DOC) [file pgen.1002734.s003.doc]

**TABLE S1.**

| Chr | Start (hg 19) | End (hg 19) | Size (bp) | Type | Genes disrupted by breakpoints | Other duplicated/  deleted genes in the region | Cohort observed, age ^a^ |
| --- | --- | --- | --- | --- | --- | --- | --- |
| **Cases only** | |  |  |  |  |  |  |
| 1 | 158,553,308 | 158,698,075 | 144768 | dup | - | *OR10Z1, OR6K2, OR6K3, SPTA1* | Fam, 54 |
| 2 | 36,883,075 | 37,087,940 | 204866 | dup | *STRN* | *VIT* | Fam, 58  Ybr, 38 |
| 2 | 197,360,005 | 197,539,960 | 179956 | dup | *CCDC150, HECW2* | *-* | Fam, 48  Fam, 63+70 |
| 2 | 238,192,383 | 238,556,182 | 363780 | dup | *LRRFIP1* | *COL6A3, MLPH, RAB17* | Ybr, 38 |
| 3 | 37,747,296 | 37,807,058 | 59763 | del | *ITGA9* |  | Fam, 37 |
| 3 | 193,864,987 | 194,398,868 | 533881 | dup | *-* | *ATP13A3, CPN2, GP5, LOC100131551, LRRC15, LSG1* | Fam, 43+53 |
| 4 | 17,346,427 | 17,630,260 | 283834 | dup | *-* | *CLRN2, LAP3, MED28, QDPR* | Ybr, 40 |
| 4 | 20,640,647 | 20,749,299 | 108653 | dup | *KCNIP4* | *PACRGL* | Fam, 70 |
| 4 | 185,507,794 | 185,840,382 | 332589 | del | *-* | *ACSL1, CASP3, CCDC111, MLF1IP, SLED1* | Fam, 50 |
| 5 | 10,361,110 | 10,941,988 | 580879 | dup | *MARCH6* | *ANKRD33B, ROPN1L, DAP* | Fam, 50 |
| 6 | 65,720,728 | 66,026,033 | 305306 | del | *EYS* | *-* | Fam, 53 |
| 7 | 8,376,567 | 8,481,796 | 105230 | del | *NXPH1* | *-* | Ybr, 38 |
| 8 | 53,434,613 | 53,809,456 | 374844 | dup | *-* | *RB1CC1, FAM150A* | Fam, 37 |
| 8 | 118,974,006 | 119,112,045 | 138040 | del ^c^ | *EXT1* | *-* | Ybr, 32 |
| 8 | 141,461,164 | 141,598,282 | 137119 | dup | *EIF2C2, TRAPPC9* | *CHRAC1* | Ybr, 31 |
| 8 | 145,725,796 | 145,756,558 | 30763 | del | *ARHGAP39, PPP1R16A* | *GPT, LRRC14, LRRC24, MFSD3, MGC70857, RECQL4* | Fam, 89+ovca 50 |
| 9 | 73,718,807 | 74,354,983 | 636177 | dup | *TMEM2, TRPM3* | *-* | Ybr, 39 |
| 9 | 124,240,146 | 124,344,069 | 103924 | del | *DAB2IP, GGTA1* | *-* | Ybr, 39 |
| 10 | 14,986,306 | 15,065,749 | 79444 | del | *DCLRE1C* | *MEIG1* | Fam, 47 |
| 12 | 100,275,135 | 100,462,609 | 187474 | dup | *ANKS1B, UHRF1BP1L* | *-* | Fam, 40  Fam, 45  Fam, 54 |
| 13 | 70,652,255 | 70,793,725 | 141471 | del | *KLHL1* | *ATXN8OS* | Fam, 39 |
| 14 | 64,537,498 | 64,768,302 | 230805 | dup | *ESR2, SYNE2* | *-* | Fam, 72  Ybr, 40 |
| 15 | 90,753,755 | 91,260,219 | 506465 | dup ^d^ | *SEMA4B, BLM* | *CIB1, c15orf58, TTLL13, NGRN, GABARAPL3, ZNF774, CRTC3, IQGAP1* | Fam, 39+45 |
| 16 | 7,739,518 | 7,848,144 | 108627 | dup | *RBFOX1* | *-* | Fam, 45 |
| 16 | 78,018,616 | 78,071,701 | 53086 | del | *-* | *CLEC3A* | Ybr, 39 |
| 18 | 29,734,595 | 29,806,705 | 72111 | del | *-* | *MEP1B* | Ybr, 39 |
| 21 | 18,776,281 | 19,032,879 | 256599 | dup | *-* | *CXADR, BTG3* | Fam, 71 |
| **Controls only** | |  |  |  |  |  |  |
| 5 | 130,626,179 | 130,830,528 | 204350 | del | *CDC42SE2, RAPGEF6* | *-* | Healthy, 61 ^b^ |
| 6 | 33,114,409 | 33,139,835 | 25427 | dup | *COL11A2* | *-* | Healthy, 54 |
| 6 | 118,654,228 | 119,106,298 | 452071 | dup | *-* | *BRD7P3, C6orf204, PLN* | Healthy, 56 |
| 6 | 142,714,201 | 142,929,817 | 215617 | del | *GPR126, LOC153910* | *-* | Healthy, 57 |
| 7 | 51,097,894 | 51,177,432 | 79539 | del | *COBL* | *-* | Healthy, 55 |
| 8 | 6,437,580 | 6,489,978 | 52399 | del | *MCPH1* | *-* | Healthy, 59 |
| 12 | 1,139,147 | 1,340,614 | 201468 | dup | *ERC1* | *-* | Healthy, 66 |
| 14 | 96,618,708 | 96,741,342 | 122635 | dup | *-* | *BDKRB1, BDKRB2* | Healthy, 57 |
| 16 | 9,091,342 | 9,355,611 | 264270 | dup | *-* | *C16orf72, MIR548X* | Healthy, 64  Healthy, 54 |
| 16 | 58,443,325 | 58,496,970 | 65801 | del ^e^ | *NDRG4* |  | Healthy, 65 |
| 16 | 84,469,311 | 84,583,727 | 114417 | dup | *ATP2C2* | *KIAA1609* | Healthy, 61 ^b^ |
| 16 | 84,774,123 | 84,946,428 | 172306 | dup | *USP10* | *CRISPLD2* | Healthy, 56 |
| 17 | 64,410,125 | 64,486,135 | 76011 | Hom. del ^c^ | *PRKCA* |  | Healthy, 54 |
| 20 | 2,077,693 | 2,161,599 | 83907 | dup | *-* | *STK35* | Healthy, 50 |
| 21 | 32,711,357 | 32,917,773 | 206417 | del | *TIAM1* |  | Healthy, 50 |
| X | 13,049,926 | 13,629,522 | 579596 | dup | *EGFL6* | *FAM9C, ATXN3L* | Healthy, 54 |
| X | 5,737,686 | 6,442,425 | 704740 | dup | *-* | *NLGN4X* | Healthy, 54 |

Chr = chromosome; del = deletion; dup = duplication; hg 19 = human genome assembly 19 (February 2009); hom = homozygous; ovca = ovarian cancer

^a^ Fam = familial breast cancer case; Ybr = young breast cancer case. Age = age at diagnosis, or age at monitoring for healthy controls

^b^ Same individual

^c^ Intronic

^d^ Breakpoint <1 kb from *BLM* 5’ end, interrupts the promoter

^e^ Breakpoint exactly (0.7 kb) at 5' end of *NDRG4* interrupting the promoter and 3 kb after 3' end of *GINS3*
