# Supplementary material for: Rare Copy Number Variants Observed in Hereditary Breast Cancer Cases Disrupt Genes in Estrogen Signaling and TP53 Tumor Suppression Network
Source: PLoS Genet. 2012 Jun 21;8(6):e1002734. doi: 10.1371/journal.pgen.1002734 (PMC3380845; doi:10.1371/journal.pgen.1002734)
Supplement: Table S2 — Novel rare CNVs in genomic DNA that delete or duplicate genes observed in both breast cancer cases and controls. (DOC) [file pgen.1002734.s004.doc]

**TABLE S2.**

| Chr | Start (hg 19) | End (hg 19) | Size (bp) | Type | Genes disrupted by breakpoints | Other duplicated/  deleted genes in the region | Cohort observed ^a^ |
| --- | --- | --- | --- | --- | --- | --- | --- |
| 1 | 12,332,721 | 12,778,440 | 445720 | dup | *AADACL3,*  *VPS13D* | *AADACL4, DHRS3, SNORA59A, SNORA59B* | Ybr 2/75  Cont 1/128 |
| 6 | 161,351,012 | 161,552,676 | 201665 | dup | *AGPAT4* | *MAP3K4* | Ybr 1/75  Cont 2/128 |
| 10 | 51,827,677 | 52,161,557 | 333881 | dup | *SGMS1* | *ASAH2, FAM21A, FAM21B* | Fam 1/103  Ybr 2/75  Cont 1/128 |
| 10 | 96,497,371 | 96,558,977 | 61607 | del | *CYP2C19* | *-* | Fam 6/103  Ybr 1/75  Cont 3/128 |
| 10 | 81,411,656 | 82,023,440 | 611785 | dup | *-* | *ANXA11, C10orf57, LOC100288974, LOC219347, LOC642361, LOC650623, MBL1P, PLAC9, SFTPD* | Fam 1/103  Cont 1/128 |
| 18 | 64,081,296 | 64,339,529 | 258234 | del | *-* | *CDH19* | Fam 1/103  Ybr 1/75  Cont 1/128 |

Chr = chromosome; del = deletion; dup = duplication; hg 19 = human genome assembly 19 (February 2009)

^a^ Fam = familial breast cancer case; Ybr = young breast cancer case; Cont = healthy control
