# Supplementary material for: Rare Copy Number Variants Observed in Hereditary Breast Cancer Cases Disrupt Genes in Estrogen Signaling and TP53 Tumor Suppression Network
Source: PLoS Genet. 2012 Jun 21;8(6):e1002734. doi: 10.1371/journal.pgen.1002734 (PMC3380845; doi:10.1371/journal.pgen.1002734)
Supplement: Table S3 — Novel rare CNVs in genomic DNA that delete or duplicate genomic regions without annotated genes in breast cancer cases and controls. (DOC) [file pgen.1002734.s005.doc]

**TABLE S3.**

| Chr | Start (hg 19) | End (hg 19) | Size (bp) | Type | Flanking genes (distance, 3’/5’end of the gene facing the CNV ^a^) | Cohort observed, age ^b^ |
| --- | --- | --- | --- | --- | --- | --- |
| **Cases only** | |  |  |  |  |  |
| 5 | 55,697,237 | 56,021,536 | 324300 | dup | *ANKRD55* (170 kb, 5’), *MAP3K1* (90 kb, 5’) ^c^ | Fam, 26 |
| 8 | 123,500,790 | 123,568,747 | 67958 | dup | *ZHX2* (229 kb, 5’) | Fam, 55  Ybr, 40 |
| 11 | 29,595,051 | 29,718,369 | 123319 | del | *KCNA4* (139 kb, 5’) | Fam, 56 |
| 11 | 42,913,491 | 42,970,764 | 57274 | dup | *API5* (365 kb, 5’) | Ybr, 40 |
| 12 | 83,535,046 | 85,079,347 | 1544302 | del | *TMTC2* (35 kb, 3’), *SLC6A15* (198 kb, 5’) | Ybr, 34 |
| 13 | 84,541,089 | 84,678,285 | 137197 | del | *SLITRK1* (86 kb, 5’) | Fam, 89 |
| 15 | 36,123,226 | 36,187,085 | 63860 | del | *ATPBD4* (287 kb, 5’) | Ybr, 40 |
| 17 | 13,156,796 | 13,235,925 | 79130 | dup | *HS3ST3A1* (165 kb, 3’) | Ybr, 40 |
| 18 | 64,816,997 | 65,011,782 | 194786 | dup | *DSEL* (164 kb, 5’), *CDH19* (546 kb, 5’) | Fam, 50 |
| X | 121,301,574 | 121,481,602 | 180029 | dup | *GRIA3* (849 kb, 5’) | Ybr, 35 |
| **Controls only** | |  |  |  |  |  |
| 1 | 187,991,293 | 188,270,238 | 278946 | del | *c1orf99* (380 kb, 5’) | Healthy, 59 |
| 2 | 193,671,303 | 193,858,558 | 187256 | del | *PCGEM1* (31 kb, 3’) | Healthy, 65 |
| 4 | 59,482,852 | 59,670,031 | 187180 | del | none within 1200 kb | Healthy, 51 |

Chr = chromosome; del = deletion; dup = duplication; hg 19 = human genome assembly 19 (February 2009)

^a^ Orientation of the gene towards the breakpoint is shown 3’ = 3’ end of the gene is adjacent to breakpoint; 5’ = 5’ end of the gene is adjacent to breakpoint

^b^ Fam = familial, Ybr = young breast cancer case. Age = age at diagnosis, or age at monitoring for healthy controls

^c^ This genomic area locates near hit in GWAS, mediated by rs889312 SNP [3]
